# Characterization of *Eucalyptus nitens* Lignins Obtained by Biorefinery Methods Based on Ionic Liquids

**DOI:** 10.3390/molecules25020425

**Published:** 2020-01-20

**Authors:** Lucía Penín, Heiko Lange, Valentín Santos, Claudia Crestini, Juan Carlos Parajó

**Affiliations:** 1 Chemical Engineering Department, Polytechnical Building, University of Vigo (Campus Ourense), As Lagoas, 32004 Ourense, Spain; lpenin@uvigo.es (L.P.); vsantos@uvigo.es (V.S.); 2 Department of Pharmacy, University of Naples ‘Federico II’, Via Domenico Montesano, 49, 80131 Naples, Italy; heiko.lange@unina.roma2.it; 3 Department of Molecular Sciences and Nanosystems, University of Venice ‘Ca’Foscari’, Via Torino 155, 30170 Venice Mestre, Italy

**Keywords:** *Eucalyptus nitens*, fractionation, ionic liquids, lignin, ^31^P-NMR, HSQC

## Abstract

*Eucalyptus nitens* wood samples were subjected to consecutive stages of hydrothermal processing for hemicellulose solubilization and delignification with an ionic liquid, i.e., either 1-butyl-3-methylimidazolium hydrogen sulfate or triethylammonium hydrogen sulfate. Delignification experiments were carried out a 170 °C for 10–50 min. The solid phases from treatments, i.e., cellulose-enriched solids, were recovered by centrifugation, and lignin was separated from the ionic liquid by water precipitation. The best delignification conditions were identified on the basis of the results determined for delignification percentage, lignin recovery yield, and cellulose recovery in solid phase. The lignins obtained under selected conditions were characterized in deep by ^31^P-NMR, ^13^C-NMR, HSQC, and gel permeation chromatography. The major structural features of the lignins were discussed in comparison with the results determined for a model Ionosolv lignin.

## 1. Introduction

Lignocellulosic materials represent a widespread, low cost and renewable bioresource suitable as a raw material for the sustainable production of fuels, chemicals and materials. In quantitative terms, woods are the most important type of lignocellulosic materials. Considered as feedstocks for the industry, woods show important comparative advantages over other lignocellulosic materials of agricultural origin, including large availability, non-seasonal character, favorable composition, and ability to grow in lands unsuitable for agriculture (avoiding direct and indirect competition with the food chain).

In typical cases, about 90% of the wood weight correspond to the structural components, which include polysaccharides, i.e., cellulose and hemicelluloses, and lignin, an amorphous and aromatic biosynthesized polymer.

*Eucalyptus* spp. are fast growing hardwoods with favorable features to be valorized by sustainable conversion technologies [1]. *Eucalyptus* is the most widely planted type of hardwoods and can be produced at relatively low cost [2]. Although the most important *Eucalyptus* species in terms of world plantations are *E. grandis*, *E. urophylla*, *E. camaldulensis*, and *E. globulus*, growing attention is being paid to *E. nitens*, owing to their decreased susceptibility to *Gonipterus* plagues and to its ability to resist a wide range of environmental conditions, i.e., altitudes between 600 and 1600 m, with moderate temperatures in summer and cold temperatures, frost and snow in winter [3,4]. Because of this, *E. nitens* is an interesting raw material for lignocellulose biorefineries, conceived as integrated industrial facilities enabling the separation of the major feedstock components and their separate processing. 

The fractionation of the raw materials according to the biorefinery concept can be achieved by diverse technologies. In the past few years, ionic liquids (ILs) have emerged as alternative agents for physical separation and/or chemical transformation of lignocellulosic materials in biorefineries [5].

Ionic liquids (ILs) present low melting temperatures, very low volatility, and good thermal and chemical stabilities. Owing to these properties, ILs have been considered environmentally friendly materials for the fractionation of lignocellulosic biomass [6,7].

ILs are usually classified according the type of cation. The most suited ones for *Eucalyptus* wood fractionation bear the methylimidazolium cation: studies have been reported on the utilization of several ILs of this type, i.e., 1-allyl-3-methylimidazolium chloride, 1-butyl-3-methylimidazolium acesulfamate, 1-butyl-3-methylimidazolium acetate and 1-ethyl-3-methylimidazolium acetate for processing woods of *E. nitens*, *E. globulus*, *E. grandis*, and unidentified species [8,9,10,11].

One of the possible targets in the IL-mediated fractionation of wood is the isolation of lignin. To our knowledge, the only study on the delignification of *E. nitens* wood with ILs has been reported by Pinkert et al. [9], who employed 1-butyl-3-methylimidazolium acesulfamate to isolate lignin fractions that were characterized by IR spectroscopy, elemental analysis, and thermal methods. 

In the last years, the application of NMR-based methods has enabled a deeper understanding on the structure and chemistry of lignins: ^31^P-NMR enables the identification of the functional groups present in lignins [12], whereas the Heteronuclear Single Quantum Coherence spectroscopy (HSQC) allows the study of the aromatic subunits and the degree of condensation, as well as the presence of ether bonds [13,14].

As a typical hardwood, *E. nitens* possess a lignin mainly made up of syringyl units. Milled wood lignin from *E. nitens* has been reported to show a predominance of β-*O*-4′ ether linkages, followed by β-β′ resinol-type linkages and lower amounts of β-5′ phenylcoumaran-type and β-1′ spirodienone-type linkages [15]. When the lignin is fractionated from lignocellulose for further characterization, the isolation method affects the properties of the samples, also in the case of IL-mediated approaches. Besides the study of Pinkert et al. [9] cited above, experimental information has been reported on the characterization of *E. nitens* lignins isolated in media containing concentrated acetic acid [16].

**Figure 1:**
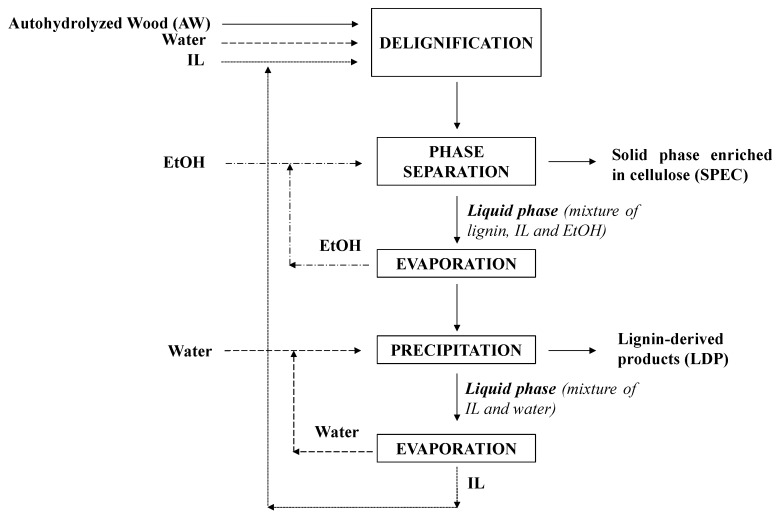
General scheme followed for lignin separation.

Obtaining added value from lignin is a key requirement for the implementation lignocellulose biorefineries. This work deals with a complete fractionation of *E. nitens* wood, which enabled the production of highly functionalyzed, sulfur-free, soluble, reactive lignin potentially suitable for applications in a number of fields highlighted in literature [17,18,19], including materials and polymers, such as carbon fibers, dispersants, emulsifiers, chelating agents, adsorbents, adhesives, composites, and resins, transformation by depolymerization into simple phenols, including aromatic acids, alcohols, esters, ethers, and aldehydes, or a feedstock for producing specialty and commodity products, including hydrocarbon fuels. In summary, our study provides a sound assessment on a complete fractionation method, based on two reaction steps (hemicellulose solubilization in aqueous media and delignification in ionic liquids). This operational scheme allowed an extensive and selective separation of fractions, which are suitable for specific applications, according to the biorefinery philosophy. The complete set of experimental data allowed the formulation of material balances, and the chemical structure of lignin-derived products is assessed in deep. Specifically, *E. nitens* wood was first subjected to hydrothermal processing to achieve the selective solubilization of hemicelluloses, leaving solid phase with increased contents of cellulose and lignin. Delignification of this solid substrate yielded a solid enriched in cellulose, and a liquid phase containing the IL and soluble lignin fragments. The hydrothermal treatment was performed under optimal conditions identified in a previous study [16], and the delignification of the hemicellulose-depleted solid (denoted autohydrolyzed wood, AW) was performed in media containing an ionic liquid, 1-butyl-3-methylimidazolium hydrogen sulfate, denoted [bmim]HSO_4_, or triethylammonium hydrogen sulfate, denoted [TEA]HSO_4_. Figure 1 summarizes the method for lignin separation considered in this study. After delignification, the IL-rich media were mixed with ethanol to facilitate the separation of the cellulosic phase by centrifugation. After ethanol recovery by evaporation, the lignins were separated from the reaction media by precipitation with water, and characterized by ^31^P-NMR, ^13^C-NMR, HSQC and Gel Permeation Chromatography (GPC), to obtain key information defining their potential as substrates for further chemical conversion into added-value products. Quantitative data concerning the yields and compositions of the various phases involved in the considered processing scheme are also provided. The results are discussed in comparison with the results obtained for an Ionosolv pine lignin obtained under optimal fractionation conditions [20].

## 2. Results and Discussion

### 2.1. Wood Composition

The *E. nitens* wood lot employed in experiments was the same employed in a previous article [21] and showed the following contents of the structural components (as wt%): cellulose, 42%; hemicelluloses, 22.3% and Klason lignin, 21.4%. Other components appearing in minor amounts were: ash, 0.25%; extractives, 4.7%; and acid soluble lignin, 1.5%.

### 2.2. Wood Autohydrolysis

The aqueous fractionation of wood was performed under non-isothermal conditions reaching up to 195 °C, the temperature at which the maximum conversion of xylan into soluble saccharides was achieved [21]. The combined effects of temperature and time during the reaction were assessed in terms of severity as defined by Overend and Chornet [22]:

(1) $$\mathrm{Severity}=\log\left(\int_{T_{0}}^{T_{\max}}e^{\frac{T\left(t\right)-T_{\mathrm{ref}}}{T_{\omega }}}\cdot \mathrm{dt}\right)$$

where T_0_ is the initial temperature, T_max_ the maximum temperature, T(t) the temperature profile and T_ref_ the reference temperature (100 °C), while the parameter T_ω_ takes the value 14.75 °C. Using this equation, the severity calculated for the experiment performed at 195 °C (including the heating and cooling periods) was 3.62. Operating under these conditions, the autohydrolyzed wood (AW), was recovered at 72.8% solid yield, while 80.7% xylan was solubilized. The compositions of the solid and liquid phases from autohydrolysis processing are the same detailed in Penín et al. [21]. 

### 2.3. Ionic Liquid (IL) Delignification: Lignin Removal and Recovery

In order to achieve the separation of lignin from cellulose, AW was subjected to delignification in ionic liquid (IL) in the presence of 20% water (measured respect to the solid mass). Two ionic liquids ([bmim]HSO_4_ and [TEA]HSO_4_) were used as delignification agents. [bmim]HSO_4_ has been used to produce furfural from C5 saccharides [23,24], evidencing that the acidic character of [bmim]HSO_4_ enabled a double role as a catalyst and as a solvent, a behavior potentially useful for achieving the delignification of lignocellulosic materials. In comparison, [TEA]HSO_4_ is a low cost ionic liquid that shows comparative advantages respect to other ILs in terms of dissolution ability and thermal stability [25]. 

Preliminary experiments (data not shown) were performed at 160–180 °C. The maximum delignification percentage of was achieved operating at 180 °C, but the selectivity was poor owing to increased cellulose losses. Because of this, 170 °C was chosen as compromise temperature enabling both a satisfactory reaction rate and a high cellulose recovery in solid phase, and the reaction time was considered as an operational variable for optimization. 

**Figure 2:**
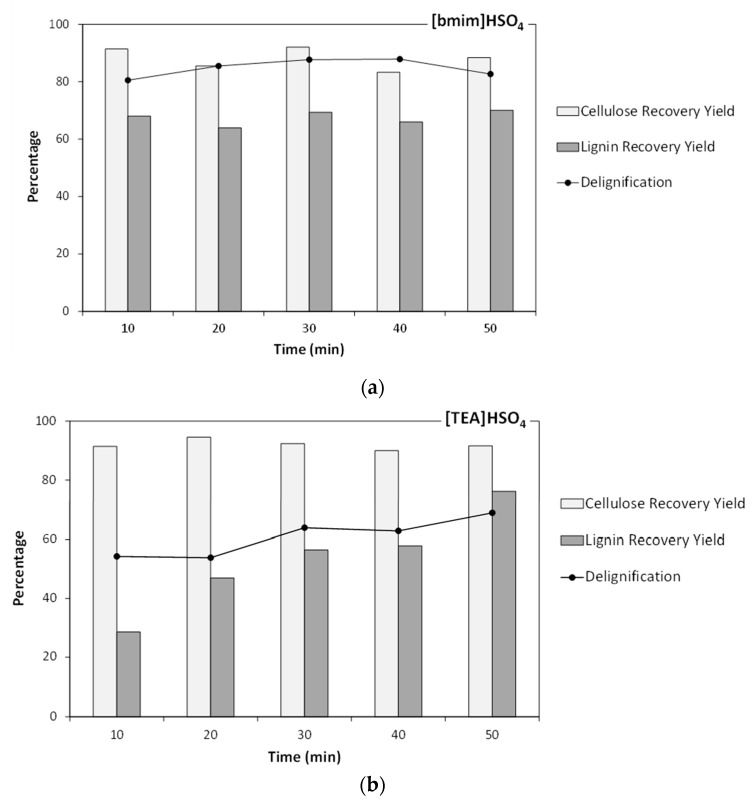
Results obtained in delignification treatments carried out at 170 °C using: (**a**) [bmim]HSO_4_, and (**b**) [TEA]HSO_4_.

Figure 2 shows the experimental results obtained in treatments at 170 °C with both ILs. In experiments using [bmim]HSO_4_ (Figure 2a), the degree of delignification reached 87.9% in the experiment lasting 40 min, but the percentage of cellulose recovery dropped considerably (83%) with respect to treating the sample under these conditions for only 30 min (92%). The percentage of delignification reached 87.7% in the experiment lasting 30 min, and then remained almost unchanged, indicating a fast reaction rate. These results were in the range reported by Xu et al. [26] for the delignification of *Tamarix austromongolica* using the ionic liquid [bmim]Ace (50% delignification after 3 hours), and Brandt et al. [27] (90% delignification after 22 h).

Lower delignification degrees were achieved with the ionic liquid [TEA]HSO_4_ (see Figure 2b). The maximum delignification percentage was 69% (reached after 50 min), similar to the result reported by Li et al. [28] (69% lignin removal after 3 h). However, this IL enabled high cellulose recoveries, which remained fairly constant (90–95%) along the experimental domain.

The yields and compositions of the solid phases obtained after delignification are shown in Table 1. Low contents of residual xylan were observed in all cases, with tendency to decrease with the reaction time. No arabinan was present, and the contents of acetyl groups were negligible. The cellulose contents increased along treatments, reaching 80–88% in assays with [bmim]HSO_4_ and 72–77% in assays with [TEA]HSO_4_. In [bmim]HSO_4_ media, the Klason lignin content presented a minimum value (5.9% after 30 min). The observed dependence of the Klason lignin content with the reaction severity (measured by the reaction time) suggested that lignin is first dissolved, and then the soluble lignin fragments underwent repolymerization, according to a mechanism of two reactions in series: under low severity conditions (in our case, short reaction times), lignin solubilization is the major effect); but in further reaction stages, the increase in the concentration of soluble lignin fragments resulted in increased lignin repolymerization, which becomes the predominant reaction. This behavior is in agreement with literature reported for lignocellulose delignification in media made up of ionic liquids [29,30].

In comparison, lower delignification degrees were reached with [TEA]HSO_4_ (minimum lignin content 13.2%, reached after 50 min).

### 2.4. Lignin Characterization

*E. nitens* lignin samples isolated under the optimal delignification conditions identified above were characterized for structural features and molecular mass aspects. Additionally, to assist in the comparison of the identified biomass fractionation methods between hardwoods and softwoods, experimental data were also recorded for an Ionosolv pine lignin (IP) isolated in [bmim]HSO_4_/water media under conditions reported as optimal [20] (170 °C, 90 min, 5:1 liquid to solid mass ratio). The diverse lignin samples were structurally analyzed using the following techniques: quantitative ^31^P-NMR, qualitative HSQC and ^13^C-NMR [12,31]. The results are presented in Table 2, Table 3 and Table 4, respectively. While ^31^P-NMR is best for both qualitatively and quantitatively analysing the OH-group contents, HSQC is an established technique for evaluating complex structural motifs in lignin and ^13^C-NMR serves to identify groups not visible in the HSQC, while exhibiting the possibility to validate HSQC interpretations, and vice versa. Additionally, gel permeation chromatography (GPC) was used to determine the molecular weight distribution of the lignins under study (see data in Table 5).

The OH-group analysis via quantitative ^31^P-NMR [12] essentially reveals that the two different IL-preparations differ significantly only in the amount of aliphatic OH-group content. In the case of [TEA]HSO_4_, intensity of the signal attributable to aliphatic OH groups is significantly stronger. In terms of phenolic end groups, no meaningful differences between [bmim]HSO_4_ and [TEA]HSO_4_ preparations were found in the phenolic OH-group content. Differences between the aliphatic OH-group content of the two samples can arise either from an insufficient cellulose separation in the case of [TEA]HSO_4_, leaving behind carbohydrates that cause an artificial increase in aliphatic OH-group content, or from a partial degradation of the phenylpropanoid structure, leading to a loss in aliphatic OH-groups along the chain in case of [bmim]HSO_4_. 

**Figure 3:**
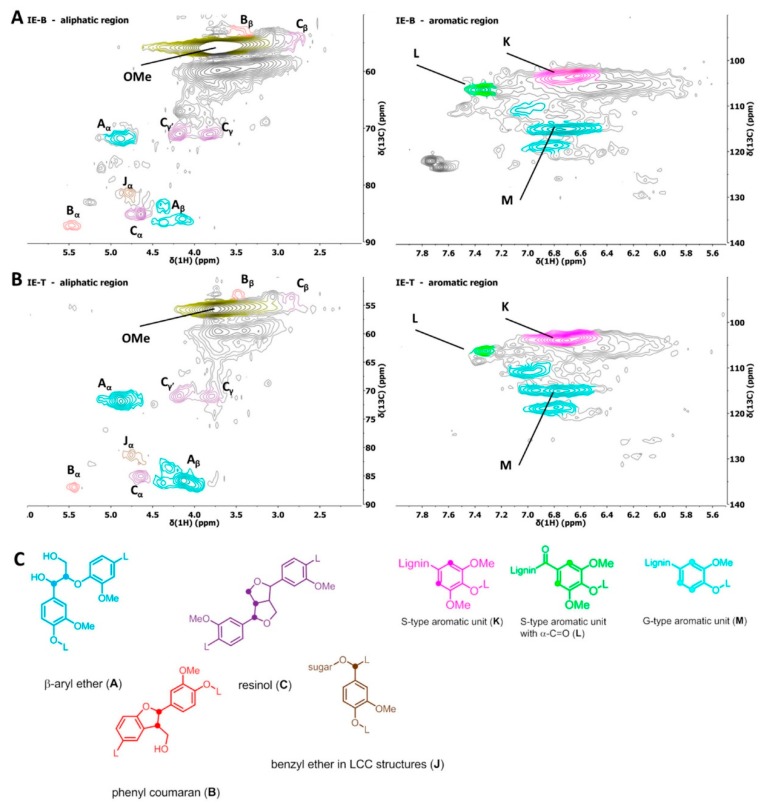
HSQC spectra of (**A**) **IE_B_** and (**B**) **IE_T_**. The aliphatic region is shown on the left, while the aromatic region is displayed on the right. (**C**) Important identified and colour-coded structural units.

For identifying the more likely reason of the aliphatic OH-group difference, comparative HSQC analysis was performed, using a standard, non-quantitative HSQC sequence, keeping sample concentration and acquisition parameters constant. The results are given in Table 5, whereas the HSQC spectra are shown in Figure 3.

A detailed analysis and comparison of the various NMR data generated confirmed the presence of the groups indicated in Table 3. Analysis of the identified structural motifs suggests that both ionic liquid treatments were efficiently, albeit not fully removing cellulose and hemicellulose. Only traces of carbohydrate impurities are present in comparable amounts in both samples, comprising a weak signal attributable to benzyl ether motifs claimed in lignin-carbohydrate complexes (LCCs) [32]. Data obtained from ^31^P-NMR (Table 2) show a low content in aliphatic OH groups and a very high content in phenolic groups with respect to data previously reported for milled wood lignins [13]. In all cases, significant amounts of condensed groups, i.e., non-etherified 5-5′, 4-*O*-5′ and diphenylmethane are present in the isolated lignins. This indicates that all the considered treatments induce significant structural modifications in the native lignin backbone, since these units are absent in milled wood lignins. The generation of condensed units upon acidic fractionation conditions has been previously reported in the literature [33]. The ratio of condensed units to total phenolic content in the two eucalyptus lignin samples, however, is not significantly different thus implying a similar behavior of the two different ionic liquids examined. The phenolic to aliphatic hydroxy groups ratio was found much higher in the [bmim]HSO_4_ rather than in the [TEA]HSO_4_-*E. nitens* lignin sample. Since the difference is clearly more significant than suggested by the HSQC spectra obtained, this indicates a significant decrease of the aliphatic OH groups, an increase of the phenolic units or both. 

The intensities of the signals assigned to the lignin interunit bondings as revealed by HSQC, were normalized to the signal of the methoxy groups in order to obtain a relative amount ratio. On this basis one can conclude that the use of [bmim]HSO_4_ yields an overall depletion of typical lignin interunit bonding signals with respect to [TEA]HSO_4_-*E. nitens* lignin. This implies that the lignin backbone has suffered more during the [bmim]HSO_4_ isolation process. Very severe depletion of interunit bonding motifs could be expected to lead to a larger number of unsaturated motifs within the backbone. Yet the HSQC spectra do not exhibit significant evidence for drastic degeneration. The HSQC spectrum IE_B_ does indicate, however, an elevated level of α-oxidised β-*O*-4′ units with respect to **IE_T_**. Also, this explains a reduced aliphatic OH to phenolic OH ratio. The listed observations suggest that the treatment with [bmim]HSO_4_ is overall less suitable for isolating also a higher value lignin stream.

In case of [bmim]HSO_4_, data further suggest that the washing out of the ionic liquid from the lignin was not fully successful. As focused cross peaks in the aliphatic region indicate the presence of noticeable amounts of IL, this may as such also influence the ^31^P-NMR results leading to underestimation of absolute OH-groups amount in the [bmim]HSO_4_ lignin.

Additional ^13^C-NMR analyses of the two lignins were performed to eventually support HSQC-based structural characterization, identified structural motifs are listed in Table 4. The ^13^C-NMR data support the HSQC-based conclusions: **IL_B_** ([bmim]HSO_4_-derived *E. nitens* lignin) generated signals typical for oxidized β-*O*-4′ units, while signals of interunit bonding motifs in general show lower intensities compared to [TEA]HSO_4_-*E. nitens* lignin in qualitative comparative analyses. 

Structural analyses were completed by gel permeation chromatography for molecular weight features (see Table 5). GPC data indicated that *E nitens* lignin isolated using [bmim]HSO_4_ consisted of lower molecular weight distribution than [TEA]HSO_4_-derived *E nitens* lignin; polydispersity is higher for **IE_B_** as well. This, at least partially, explains the higher phenolic to aliphatic OH ratio in this sample. These observed differences in the molecular weight can be interpreted as a confirmation of the fact that the use of [bmim]HSO_4_ leads to a more significant degradation of the lignin backbone during the fractionation process.

Comparing the structural observations generated in this study with previously reported data, it can be stated that this study is by and large in line with findings made by other groups using various ILs for fractionating lignocellulosic biomass from different hard- and softwoods. The problem is incorporation of [bmim]-type ions as impurity has been reported before [9]. Our study does not manage to delineate in a concluding fashion whether the IL impurities found in **LE_B_** resulted from residues just not washed out efficiently, or whether the observable cross-peaks resulted from eventually covalently bound former bmim ions, that under the chosen process conditions lost their chemical inertness. Brandt et al. [27] reported a relatively sluggish performance of [bmim]-type ionic liquids in general compared to other ionic liquids in terms of fractionating lignocellulosic biomass. TEA-based ionic liquids, on the contrary, have been found to work well in fractionation processes before [25]. Most noteworthy, however, is the fact that various groups observed partial lignin degradation when using acidic ILs in biomass fractionation. Varanasi et al. [10] suggested that the observed differences in the ratio between aliphatic OH-groups and phenolic OH-groups, in combination with the significantly reduced molecular weight, could be indeed attributed to a partial degradation. The fact that this is observed mainly in case of [bmim]HSO_4_ suggests a crucial role of the tight interplay between cation and anion also in biorefinery applications of ionic liquids, as hypothesized before. Given that existing reports blame IL acidity for lignin degradation, it could be speculated that the ammonium ion mitigates this acidity. More detailed studies are needed, however, to shed light into this aspect.

Comparing the results generated for the *E. nitens* hardwood with the structural data generated for the pine Ionosolv lignin (in light of both the information available for this specific case and the results reported for pine fractionation using other ionic liquids), it can be stated that [TEA]HSO_4_ yields a less degraded lignin also in the case of pine, that is not suffering from background backbone degradation or trapped IL residues. 

With respect to an eventual downstream processing of the lignin, the differing structural features of **IE_B_** and **IE_T_** might represent a valuable feature of the developed process. Given that both methods led to a by and large successful fractionation of the biomass components, assuming further that the yet insufficient removal of [bmim] from IE_B_ can be remedied in a second process optimization, the presented fractionation processes are thus valuable additional methods for eventually pre-tailoring the lignin already during its isolation for a downstream use.

## 3. Materials and Methods

### 3.1. Raw Material and Analysis

*Eucalyptus nitens* wood samples were collected locally, milled to a particle size below 1 cm, air-dried and stored for further analysis and processing as described below. Samples were assayed for moisture, extractives and quantitative acid hydrolysis using standard TAPPI or NREL methods. Klason lignin was measured as the insoluble material from the quantitative acid hydrolysis. The contents of cellulose and hemicellulose were measured from HPLC determination of the monosaccharides present in hydrolyzates. More detailed information on the methodology can be found in Penín et al. [21]. All NMR reagents were purchased from Sigma-Aldrich/Merck KGaA, Darmstadt, Germany.

### 3.2. Hydrothermal Processing of Wood and Analysis of Phases

Water and *E. nitens* wood samples were mixed at a liquid to solid ratio of 8 kg liquid/kg oven-dry wood. The mixture was heated in a 3.75 L stainless steel, pressurized reactor (Parr Instruments Company, Moline, IL) up to reach the desired temperature (195 °C), and then cooled immediately. Solid samples from the resulting solid (AW) were used for solid yield determination and analysis, using the same methods employed for the characterization of native wood. Aliquots of the liquid phases from the autohydrolysis stage were filtered through 0.45 µm membranes and analyzed by HPLC for monosaccharides, acetic acid, hydroxymethylfurfural (HMF) and furfural (F), using the same conditions specified above. 

### 3.3. Delignification with Ionic Liquids and Lignin Recovery

Delignification of autohydrolyzed wood (AW) was carried out using [bmim]HSO_4_ or [TEA]HSO_4_ in the presence of water (20% respect to the amounts of IL and solid substrate). Operation was performed in flasks immersed in an oil bath at 160–180 °C using 5:1 liquid to solid mass ratio. 96% ethanol was added to the reaction media (4:3 *w*/*w*) to facilitate the separation of the cellulose-enriched solids (SPEC, see Figure 1) by centrifugation (5000 rpm, 15 min). For purification, SPEC were washed three times with 96% ethanol and extracted in Soxhlet for 6 h before determination of yield and composition (using the same methodology as for raw material). The supernatants from centrifugation, the washing liquors, and the liquid phases from Soxhlet extraction were mixed and vacuum-evaporated at 40 °C, leaving an ionic liquid-lignin solution, from which lignin was precipitated by adding water (3 g water/g medium). The precipitated solids (lignin-derived products, LDP) were separated by filtration, washed with water and dried, as shown in Figure 1. Water was removed from the IL phase by evaporation for IL recovery.

### 3.4. Variables Employed to Characterize the Separation and Recovery of Components 

The variables employed and their definitions are as follows:

(2) $$\mathrm{Cellulose}\;\mathrm{recovery}\;\mathrm{yield},\;\mathrm{CRY}=\;\frac{\mathrm{Cellulose}\;\mathrm{in}\;\mathrm{SPEC}}{\mathrm{Cellulose}\;\mathrm{in}\;\mathrm{AW}}\cdot 100$$

(3) $$\mathrm{Delignification}\;\mathrm{percentage},\;\mathrm{Del}\%=\;100-\frac{\mathrm{Lignin}\;\mathrm{in}\;\mathrm{SPEC}}{\mathrm{Lignin}\;\mathrm{in}\;\mathrm{AW}}\cdot 100$$

(4) $$\mathrm{Lignin}\;\mathrm{recovery}\;\mathrm{yield},\;\mathrm{LRY}=\;\frac{\mathrm{LDP}\;\mathrm{mass}}{\mathrm{Lignin}\;\mathrm{in}\;\mathrm{AW}}\cdot 100$$

The obtained LDP were further characterized as described below. For IL recovery, water was removed from the liquid phase by evaporation.

### 3.5. Lignin Characterization by Nuclear Magnetic Resonance (NMR)

The lignins from IL treatments were assayed using the following NMR methods:

1. *^31^P-NMR Analysis* [12]. An accurately weighed amount of lignin (approximately 30 mg) was dissolved in 425 μL of anhydrous CDCl_3_/pyridine solution (1:1.6 (*v*/*v*)). 100 μL of a standard solution of cholesterol (0.1 M in anhydrous CDCl_3_/pyridine solution) containing Cr(III) acetylacetonate as the relaxation agent was added. Finally, 75 μL of 2-chloro-4,4,5,5-tetramethyl-1,3,2-dioxaphospholane (Cl-TMDP, 95%, Sigma-Aldrich) was added, and the mixture was stirred at room temperature for 2 h. The mixture was then transferred into 5 mm NMR tubes, and the spectra were measured on a Bruker (Billerica, MA, USA) AVANCE 400 MHz spectrometer equipped with a 5 mm double resonance broadband BBI inverse probe (256 scans at 20 °C), operated by Topspin software (Version 3.5). All chemical shifts reported are relative to the reaction product of water with Cl-TMDP, which gives a sharp signal in pyridine/CDCl_3_ at 132.2 ppm. NMR data were processed with MestreNova (Version 8.1.1, Mestrelab Research, Santiago de Compostela, Spain).
2. *HSQC Measurements.* Samples of around 50 mg were dissolved in 600 μL DMSO-*d*_6_ (providing NMR sample solutions with concentrations of around 83 mg/mL); chromium acetyl acetonate was added as spin-relaxing agent at a final concentration of ca. 1.5–1.75 mg/mL. HSQC spectra were recorded at 303 K on a Bruker (Billerica, MA, USA) AVANCE 400 MHz spectrometer equipped with a 5 mm double resonance broadband BBI inverse probe operated by TopSpin software (Version 3.5). The Bruker hsqcetgp pulse program in DQD acquisition mode was used, with NS = 32; TD = 2048 (F2), 512 (F1); SW = 15.0191 ppm (F2), 149.9819 ppm (F1); O2 (F2) = 2000.65 Hz, O1 (F1) = 7545.96 Hz; D1 = 2 s; CNST2 (^1^J(C–H) = 145; acquisition time F2 channel = 85.1968 ms, F1 channel = 8.4818 ms; pulse length of the 90° high power pulse P1 was optimised for each sample. NMR data were processed with MestreNova (Version 8.1.1, Mestrelab Research, Santiago de Compostela, Spain).
3. *^13^C-NMR Measurements.* Samples of approx. 80 mg of lignin were dissolved in 550 μL DMSO-*d*_6_; 50 μL chromium acetyl acetonate in DMSO-*d*_6_ (approx. 1.5 mg mL^−1^) were added as a spin-relaxation agent; 50 μL of trioxane in DMSO-*d*_6_ (approx. 15 mg mL^−1^) was used as an internal standard. The spectra were recorded at room temperature on a Bruker (Billerica, MA, USA) AVANCE 400 MHz spectrometer equipped with a 5 mm double resonance broadband BBI inverse probe operated by TopSpin software (Version 3.5). An inverse-gated proton decoupling pulse sequence was applied with a 90° pulse width, a relaxation delay of 1.7 s and an acquisition time of 1.2 s. A total of 20000-24000 scans were acquired for each spectrum. NMR data were processed with MestreNova (Version 8.1.1, Mestrelab Research, Santiago de Compostela, Spain).

### 3.6. Lignin Characterization by Gel Permeation Chromatography (GPC) 

A previously reported protocol was applied [34]: approximately 5 mg of lignin was suspended in 1 mL glacial acetic acid/acetyl bromide (9:1 *v*/*v*) for 2 h. The solvent was then carefully removed *in vacuo*, and the residue was dissolved in THF and filtered over 0.45 μm syringe filter prior to injection. By means of a sample loop, aliquots of 20 μL of the filtered solutions were analyzed at a time. GPC analyses were performed using a Shimadzu (Kyoto, Japan) instrument, operated via the LabSolutions software (Version 5.42 SP3) consisting of a controller unit (CBM-20A), pumping unit (LC 20AT), degasser (DGU-20A3), column oven (CTO-20AC), diode array detector (SPD-M20A), and refractive index detector (RID-10A). Different setups comprising either two 7.5 mm × 30 mm GPC columns Agilent (Santa Clara, CA, USA) PLgel 5 μm 10000 Å, followed by Agilent PLgel 5 μm 1000 Å) or three GPC columns (the ones mentioned above plus an Agilent PLgel 5 μm 500 Å) in series were employed. HPLC grade THF (Chromasolv^®^) was used as an eluent (0.75 mL min^−1^, at 40 °C). Standard calibration was performed with polystyrene standards (Sigma-Aldrich, MW range 162–165 × 10^6^ g·mol^−1^). Final analyses of samples were performed using the intensities of the UV signal at λ = 280 nm.

## 4. Conclusions

Efficient delignification of *Eucalyptus* nitens wood was achieved a 170 °C in media containing [bmim]HSO_4_ or [TEA]HSO_4_. [bmim]HSO_4_ performed as a remarkable delignification agent, yielding cellulose-enriched pulps of very low Klason lignin contents (5.9%). However, under conditions leading to an optimal delignification, limited selectivity toward cellulose losses was achieved (cellulose recovery yield, 83%). In comparison, [TEA]HSO_4_ enabled less lignin removal (yielding samples with 13.2% Klason lignin content), but the cellulose recovery yield increased notably (90–95%).

The structural analysis of the isolated lignins shows the suitability of both ionic liquids as media for biomass fractionation. The isolated lignins are however highly degraded with respect to milder isolation processes such dioxane extraction (yielding milled wood lignin) or organosolv treatments [34]. The lignin isolated from [bmim]HSO_4_ treatments presented a lower molecular weight distribution and comparatively low content of aliphatic OH groups, a fact ascribed to a partial degradation of the backbone. Based on the experimental data resulting from chemical characterization in form of the ratio between aliphatic OH-groups and phenolic OH-groups, presence of signals typical for oxidized β-*O*-4′ units, molar mass distribution and polydispersity, confirmed that the lignin isolated from [TEA]HSO_4_ media holds a satisfactory valorization potential via chemical transformation.

## Figures and Tables

**Table 1:** Yield and composition of the solid phase enriched in cellulose obtained upon delignification (SPEC stream in Figure 1).

Ionic Liquid	Time (min)	Solid Yield (%)	Composition (kg/100 kg SPEC)				
			Glucan	Xylan	Arabinan	Acetyl Groups	Klason Lignin
[bmim]HSO_4_	10	65.7	80.5	3.0	0.0	0.2	8.7
	20	61.2	80.8	2.3	0.0	0.2	7.0
	30	61.7	86.3	2.1	0.0	0.1	5.9
	40	58.5	82.3	1.6	0.0	0.0	6.1
	50	58.2	87.7	1.8	0.0	0.1	8.7
[TEA]HSO_4_	10	73.8	71.7	3.8	0.0	0.5	18.2
	20	75.5	72.4	3.7	0.0	0.4	18.0
	30	71.3	74.9	3.5	0.0	0.3	14.9
	40	70.7	73.7	3.4	0.0	0.3	15.5
	50	69.0	76.8	3.3	0.0	0.2	13.2

**Table 2:** Results from quantitative ^31^P-NMR analysis of *E. nitens* lignin recovered after delignification treatment for both ionic liquids and the Ionosolv lignin from *Pinus pinaster* wood (**IP_B_**).

Lignin Hydroxyl Groups	δ_P_ (ppm)	Amount (mmol/g)		
		IE_B_	IE_T_	IP_B_
Aliphatic OH	149.0–146.0	1.28	3.39	1.36
Condensed 4-*O*-5′ + 5–5′	142.8–140.3	2.05	2.18	1.25
Syringyl units	143.8–142.8	1.23	1.44	-
Guaiacyl units	140.2–138.8	0.73	0.96	1.56
*p*-OH phenol	138.8–137.4	0.14	0.16	0.24
Total phenolic OH	144.3–137.4	4.15	4.74	2.75
Carboxylic OH	135.5–134.0	0.05	0.09	0.22
Total phenolic OH/Aliphatic OH		3.2	1.4	2.02
Total phenolic OH/Condensed phenolic OH		2.0	2.2	2.2

**IE_B_**—Lignin *E. nitens* [bmim]HSO_4_; **IE_T_**—Lignin *E. nitens* [TEA]HSO_4_; **IP_B_**—Lignin *Pinus pinaster* [bmim]HSO_4_.

**Table 3:** Structural units identified in qualitative ^1^H–^13^C-HSQC analyses of *E. nitens* lignin recovered after delignification treatment for both ionic liquids; for comparison, data for the Ionosolv lignin from *Pinus pinaster* wood (**IP_B_**) [20] are listed. Chemical shifts of cross peaks used for identification are indicated.

Assignment	Cross Peak (δ_C_/δ_H_) (ppm)	IE_B_	IE_T_	IP_B_
syringyl units (S)	103.4/6.70	x	x	
guaiacyl units (G)	121.9/6.69 115.7/6.72 110.8/6.97	x	x	x
β-*O*-4′ linked to S (*erythro*)	85.7/4.64	x	x	
β-*O*-4′ linked to S (*thero*)	86.6/4.14			
β-*O*-4′ linked to S with α-C=O	106.9/7.3	x	x	
β-*O*-4′ linked to G	84.8/4.38 81.7/4.77 68.0/4.84	x	x	x
ϒ-acylated β-*O*-4´	63.0/4.36			x
β-5´	87.5/5.49 62.2/3.76	x	x	x
β-β´	85.7/4.67 71.7/4.22 53.1/3.77	x	x	x
cinnamyl aldehyde	126.9/6.82			x
cinnamyl alcohol	61.2/4.09		x	x
Υ-acylated cinnamyl alcohol	60.3/4.83			x
methoxy	56.4/3.77	x	x	x

**IE_B_**—Lignin *E. nitens* [bmim]HSO_4_; **IE_T_**—Lignin *E. nitens* [TEA]HSO_4_; **IP_B_**—Lignin *Pinus pinaster* [bmim]HSO_4_.

**Table 4:** Structural units clearly identified in qualitative ^13^C-NMR analyses of *E. nitens* lignins recovered after delignification treatment for both ionic liquids, for comparison, data for the Ionosolv lignin from *Pinus pinaster* wood (**IP_B_**) [20] are listed. Chemical shifts used for identification are indicated.

Assignment	δ_C_ (ppm)	IE_T_	IE_B_	IP_B_
syringyl units (S)	106.91–103.03 134.38–134.24	x	x	
etherified syringyl units (S)	152.72–152.28 137.96 134.74	x	x	
guaiacyl units (G)	147.8 134.38–134.24 120.32 116.79–115.40 110.64	x	x	x
etherified guaiacyl units (G)	149.15 146.75 134.13	x	x	x
5-5′	143.38 131.74 126.5	x	x	x
etherified 5-5′	145.1–144.75 132.66			x
cinnamyl aldehyde	129.65	x	x	x
cinnamyl alcohol	127.79			
H units	128.71		x	
S type β-*O*-4′ with α-C=O	106.7	x	x	
G type β-*O*-4′ (*erythro* and *threo*)	85.37 83.32 71.32 60.53	x	x	
G type β-*O*-4′ with α-C=O	63.52	x		
β-1	62.21		x	x
methoxy	55.91	x	x	x
β-β′	72.5 53.67	x		
β-5′	86.87–86.05	x		
CH_3_ groups, ketones (conj.) or in aliphatic	38.36–35.67		x	x
CH_2_ in aliphatic side chain	30.7–28.63	x	x	x
Υ-CH_3_ in *n*-propyl side chain	14.14	x	x	x

**IE_B_**—Lignin *E. nitens* [bmim]HSO_4_; **IE_T_**—Lignin *E. nitens* [TEA]HSO_4_; **IP_B_**—Lignin *Pinus pinaster* [bmim]HSO_4_.

**Table 5:** Results from Gel Permeation Chromatography (GPC) analysis of *E. nitens* lignins recovered after delignification treatment for both ionic liquids and the Ionosolv lignin from *Pinus pinaster* wood (**IP_B_**) [20].

Molecular Weight	IE_B_	IE_T_	IP_B_
Mn (g/mol)	1200	1700	1500
Mw (g/mol)	4000	4700	4100
Polydispersity index (Mw/Mn)	3.3	2.8	2.7

**IE_B_**—Lignin *E. nitens* [bmim]HSO_4_; **IE_T_**—Lignin *E. nitens* [TEA]HSO_4_; **IP_B_**—Lignin *Pinus pinaster* [bmim]HSO_4_.
